# Mass Rearing and Bioecological Observations of *Eudocima phalonia* (Lepidoptera: Erebidae) in Laboratory and Field Conditions in New Caledonia

**DOI:** 10.3390/insects17060605

**Published:** 2026-06-09

**Authors:** Lise Leroy, José Brinon, Christian G. Mille, Bruno Fogliani

**Affiliations:** 1 Équipe ARBOREAL—“AgricultuRe BiOdiveRsité Et vALorisation”, Laboratoire d’Entomologie Appliquée, Station de Recherches Fruitières de Pocquereux, IAC, Institut Agronomique Néo-Calédonien, P.O. Box 32, La Foa 98880, New Caledonia; jose.brinon@iac.nc (J.B.); bruno.fogliani@unc.nc (B.F.); 2 ISEA—Institut des Sciences Exactes et Appliquées, Université de la Nouvelle-Calédonie, BP R4, Nouméa 98851, New Caledonia

**Keywords:** *Eudocima phalonia*, New Caledonia, larval polymorphism, population dynamics, lab-rearing, fruit piercing moth

## Abstract

The fruit-piercing moth, *Eudocima phalonia* (Lepidoptera: Erebidae), is a widespread pest in the Pacific region and has recently become economically important again in New Caledonia due to increasingly frequent outbreaks. Improving knowledge of its biology is essential for developing effective monitoring and management strategies. This study aimed to (i) establish a detailed laboratory rearing protocol, (ii) characterize its life cycle and reproductive traits under controlled conditions, and (iii) explore associations between larval coloration, developmental parameters, and seasonal population dynamics. Laboratory colonies were maintained over multiple generations, and field monitoring was conducted across several sites over three years. Larval colour polymorphism was documented, and statistical analyses (ANOVA and linear models) revealed significant associations between larval coloration and pupal morphological traits. However, these results should be interpreted cautiously due to unbalanced sample sizes and the observational nature of the dataset. Differences between sexes in several developmental traits were also observed. Field data showed marked seasonal variation in population abundance. This study provides a detailed rearing protocol and baseline biological data for *Eudocima phalonia* in New Caledonia, supporting future experimental and applied research.

## Simple Summary

The fruit-piercing moth (*Eudocima phalonia*) is an important pest of fruit crops in tropical regions, including New Caledonia, where it causes significant agricultural damage by feeding on ripening fruits. This study aimed to establish a laboratory rearing protocol for *E. phalonia* and to describe key aspects of its life cycle in both controlled rearing and field monitoring over a three-year period. We observed seasonal changes in larval abundance and differences in larval colour form. Overall, this study provides a reproducible rearing framework and baseline ecological data for *E. phalonia* in New Caledonia. These results provide basic knowledge and practical tools to support future studies and pest management strategies.

## 1. Introduction

The impact of climate change on insect pest communities is increasingly evident and poses serious risks to agriculture. Rising temperatures and altered precipitation patterns are known to influence insect phenology, often leading to earlier seasonal emergence, increased numbers of generations, expanded geographic ranges, and more frequent outbreak events [1,2,3,4]. The fruit-piercing moth *Eudocima phalonia* (Linnaeus) (Lepidoptera: Erebidae) is widely distributed across tropical regions and is increasingly recognized as a pest of concern under changing environmental conditions.

First recorded in New Caledonia in 1927 [5], *E. phalonia* was historically considered an occasional pest, with outbreaks occurring every four to five years [6,7]. In recent years, however, the species has caused recurrent and economically significant damage to high-value fruit crops, particularly in 2017 and 2018 [8]. Despite its growing impact, effective and accessible control methods remain limited or difficult to implement. Poisoned bait using olfactory and gustatory attractants has shown promise for Integrated Pest Management (IPM) [9,10,11,12], but no commercial formulation is currently available in New Caledonia [8,13].

A key requirement for the development of such control strategies is the ability to rear this species under controlled conditions, allowing standardized behavioral, physiological, and ecological studies. While rearing protocols are well established for genera such as Helicoverpa and Spodoptera, they remain scarce for Eudocima. Some progress has been made with *E. materna*, including pheromone identification [14], and a few studies have reported rearing of *E. phalonia* or its parasitoids under semi-natural or outdoor conditions [15,16]. However, to date, no stable laboratory rearing protocol has been established for *E. phalonia* under controlled conditions in New Caledonia [6]. This methodological gap currently limits the development of experimental studies and the implementation of applied pest management strategies.

In addition to methodological limitations, several aspects of the species’ biology and ecology remain insufficiently documented under local conditions, particularly regarding life-history traits, larval polymorphism, and seasonal population dynamics. Field observations suggest variability in larval coloration and fluctuations in population density across years, as commonly observed in Lepidoptera, but the factors underlying these patterns remain poorly understood. In particular, it remains unclear whether these variations reflect environmental influences, density-dependent processes, or intrinsic biological variability.

The objectives of this study were therefore to: (i) establish a laboratory rearing protocol for *E. phalonia* under controlled conditions; (ii) describe its life cycle, survival, and reproductive traits; and (iii) explore potential associations (without inferring causality) between larval coloration, developmental parameters, and seasonal population dynamics based on combined laboratory and field observations. This study was designed as a descriptive and exploratory approach rather than a hypothesis-driven experimental study. By combining multi-generational laboratory rearing with three years of field monitoring, this study provides a methodological framework intended to be reproducible, along with baseline ecological data that can support future hypothesis-driven and applied research on this pest species.

## 2. Materials and Methods

### 2.1. Laboratory Rearing of Eudocima phalonia

#### 2.1.1. Collection of Wild Specimens

Wild adult specimens were collected on fruits at night, and larvae were surveyed during the day at four locations in New Caledonia: Sarraméa, Tiha, Soury-Lavergne Park, and the Fruit Research Station of the Agronomic Institute of New Caledonia (IAC) in Pocquereux (La Foa). These sites were selected for their habitat diversity, proximity to forest and cultivated areas, and the presence of larval host plants, particularly Erythrina fusca (Fabaceae). Wild-caught individuals were used to establish founding laboratory populations. To limit inbreeding effects, wild individuals were periodically introduced into the colony throughout the study period (2017–2019), approximately every 2–3 generations when available.

In addition, reproductive status and oviposition behavior were monitored in a subset of wild-captured females brought to the laboratory. A total of 16 females in 2017, 16 in 2018, and 11 in 2019 were individually observed. Each female was kept in an individual cage for oviposition monitoring. Mating status was not directly observed but inferred from the presence of fertilized eggs. For each female, the total number of eggs laid (including both fertilized and unfertilized eggs) was recorded.

#### 2.1.2. Abiotic Rearing Conditions

Rearing conditions were based on previous studies on *E. phalonia* and other noctuid moths [6,15,16]. The laboratory was maintained at a temperature of 27 ± 1 °C, relative humidity of 80 ± 5%, and a photoperiod of 13:11 (light:dark). These conditions were kept constant throughout all experiments and generations, with no intentional variation in environmental parameters. Environmental parameters were monitored daily using digital thermo-hygrometers, and any deviations greater than ±2 °C or ±10% relative humidity were recorded and corrected when possible.

#### 2.1.3. Reproduction in Laboratory Conditions

A mating aviary (210 × 110 × 62 cm) was constructed to allow free flight and mating. Two *Erythrina fusca* plants (approximately 120 cm tall) with young leaves were placed inside the aviary as oviposition substrates. Fresh fruits were suspended to stimulate natural feeding and reproductive behavior. Each mating trial consisted of one female and five males (1:5 ratio), based on preliminary trials. A total of 66 females were tested across the three years (2017–2019), including 23 females in 2017, 10 in 2018, and 33 in 2019, distributed among multiple independent mating trials. Each mating group was considered an independent replicate, resulting in a total of 66 replicates across the study. Only freshly emerged adults (<24 h) were introduced into the aviary. Mating success was assessed based on the presence of fertilized eggs. For each female, reproductive parameters were recorded, including oviposition occurrence (presence or absence of egg-laying) and the number of eggs laid (including both fertilized and unfertilized eggs).

#### 2.1.4. Laboratory Rearing Protocol

Eggs were collected daily and transferred to Petri dishes until hatching. For each generation, eggs were grouped into batches of approximately 20 individuals, each batch representing one rearing unit (experimental replicate).

First instar larvae were maintained in plastic containers (6 × 15 × 10 cm) at an initial density of approximately 20 individuals per container. From the second instar onward, larvae were divided into groups of 10 and transferred to larger boxes (29 × 19 × 12 cm) to reduce density and ensure sufficient food availability. This density was maintained until the fourth instar. At the fourth and fifth instars, larvae were maintained in groups of approximately 10 individuals per container in larger boxes (32 × 25 × 13 cm), ensuring increased space per individual. This stepwise reduction in density was implemented to standardize rearing conditions across developmental stages.

Larvae were fed with fresh *Erythrina fusca* leaves collected and replaced every two days from untreated trees. Containers were cleaned and disinfected using a 0.1% bleach solution, then rinsed and air-dried every two days. Upon pupation, individuals were transferred to emergence cages (50 × 50 × 50 cm) until adult emergence. Adults were separated by sex daily and fed either fresh fruits (e.g., mango, carambola, guava, orange, mandarin) or fruit puree without additives. Food was renewed every two days to prevent fermentation. Developmental duration and survival were recorded daily for each individual within each rearing unit. Each rearing unit was considered as an independent experimental unit for statistical analyses, although individuals within a unit were not fully independent. Therefore, statistical analyses were interpreted at the level of the rearing unit when applicable. This hierarchical structure was taken into account in the interpretation of results, and statistical inferences should therefore be considered with caution.

### 2.2. Monitoring of Natural Populations

Wild larval populations were monitored at four study sites (Sarraméa, Tiha, Soury-Lavergne Park, and the Fruit Research Station of the Agronomic Institute of New Caledonia in Pocquereux, La Foa) three times per week from January to December over the period 2017–2019. Sampling consisted of standardized visual inspections of the same host plants at each site throughout the study period, with a similar number of plants inspected at each visit. At each sampling event, all visible larvae were recorded, and developmental stage and colour morph were determined for each individual. Sampling effort was kept constant across sites and dates. For analysis, data from the four sites were pooled, as the objective was to describe regional population dynamics rather than site-specific differences. A total of 543, 944, and 388 larvae were recorded across all sites in 2017, 2018, and 2019, respectively. Only data from January to July are presented, as larval abundance declined significantly after this period and became sporadic from July to December.

### 2.3. Data Analysis

#### 2.3.1. General Approach

All statistical analyses were performed using R (version 3.6.1) in RStudio, with the package *ggplot2* used for graphical representations. Prior to analysis, data distributions were tested for normality using the Shapiro–Wilk test, and homogeneity of variances was assessed using Levene’s test. Parametric tests (Student’s *t*-test, ANOVA) were applied when assumptions of normality and homoscedasticity were met. Otherwise, non-parametric tests (Wilcoxon rank-sum test) were used. Associations between categorical variables were assessed using chi-square tests. Linear models were employed to explore relationships between larval coloration and pupal traits, and to compare biological parameters (e.g., sex-related differences and larval coloration effects).

Given the partly observational and non-randomized nature of the dataset, all statistical analyses were considered exploratory, and results were interpreted with appropriate caution.

#### 2.3.2. Survival Analysis

Survival rates were calculated for each developmental stage as the proportion of individuals reaching the next stage relative to the initial number of fertilized eggs within each rearing unit. The rearing unit was used as the statistical unit for survival analyses. For interannual comparisons, survival and mortality rates were calculated based on all monitored fertilized eggs per year, corresponding to n = 425 in 2017, n = 813 in 2018, and n = 2666 in 2019. Survival was recorded daily at the individual level, allowing the calculation of stage-specific survival rates (egg hatching, larval instars, pupation, and adult emergence). For each rearing unit, survival rates are expressed as mean ± standard deviation.

#### 2.3.3. Comparison Between Sexes and Larval Polymorphism

A subset of 300 larvae was used to investigate both sex-related differences in biological parameters (development time, pupal size, and weight) and the relationship between larval coloration and pupal traits. These larvae were randomly selected from field collections across all sampling sites and dates using a random subsampling procedure to ensure representativeness of natural populations.

For the comparison between sexes, a global linear model was constructed to assess the effect of adult sex on pupal morphological and developmental traits, including pupal length, width, weight, and pupation duration. In this model, sex was used as the explanatory variable, while pupal traits served as response variables. The significance of the model was evaluated using ANOVA. When appropriate, pairwise comparisons between sexes were conducted using Student’s *t*-test (if assumptions of normality and homoscedasticity were met) or the Wilcoxon rank-sum test (otherwise). This combined approach provided both a global assessment of sex effects and a direct comparison of mean trait values between males and females.

For the analysis of larval polymorphism, the 300 pupae previously analyzed for morphological parameters were further examined to assess the influence of larval coloration on sex ratio and on the morphological and biological characteristics of *Eudocima phalonia* pupae. Larvae were categorized into colour morphs based on the classification shown in Section 3.2.1. A linear model was used to test the relationship between colour morphotype and each quantitative variable (pupal length, width, weight, and pupation time). The effect of coloration on these variables was evaluated using ANOVA. Residuals from each model were checked to ensure the assumptions were met. If a significant effect was found, a post-hoc Tukey test was performed to compare means and categorize the colour morphs into three groups (a*, a, b) based on their effect on each quantitative variable. Due to unbalanced sample sizes among morphs, the results were interpreted with caution.

## 3. Results

### 3.1. Laboratory Rearing of Eudocima phalonia

#### 3.1.1. Reproductive Success

- Reared females

In 2017, 23 females were used for mating trials, which were primarily aimed at determining the optimal sex ratio. Of these, 14 females died without laying eggs, and only four laid fertilized eggs, producing four successive generations. In 2018, 10 females were tested, resulting in four generations with four mated females, and in 2019, 33 females were tested, with 11 successfully laying fertilized eggs, resulting in 11 consecutive generations. The reproductive success rates for 2017, 2018, and 2019 were 21.7%, 40%, and 33.3%, respectively.

- Wild females

Reproductive success in wild-caught females was comparable to that of reared females in the first two years. Of the 16 wild females captured in both 2017 and 2018, five and four females, respectively, laid fertilized eggs. In 2019, 11 wild females were captured, 10 of which had already mated and laid fertilized eggs, yielding a reproductive success rate of 91%, with only one female dying without oviposition.

#### 3.1.2. Egg-Laying Potential

- Reared females

Oviposition in reared females occurred primarily during the early evening (18:00–22:00) when males were feeding on suspended fruits. Females began feeding later, before or after laying eggs. The pre-oviposition period ranged from 8 to 13 days post-emergence, for both mated and unmated females (Table 1). The number of eggs laid and the laying duration increased across the years. The proportion of fertilized eggs varied across years, reaching 26.3% in 2017, 57.4% in 2018, and 34.2% in 2019. Egg clustering accounted for 31.1%, 19.4%, and 31.1% of the total number of eggs laid by both unmated and mated females in 2017, 2018, and 2019, respectively (n = 9, 9, and 30).

- Wild females

Due to the unknown age of wild-captured females, their egg-laying profiles were more variable. Some females laid fertilized eggs the night of capture, suggesting prior mating. Others began laying within 2–6 days post-capture, while unmated females laid sterile eggs 5–7 days later. In 2019, fertilization rates reached 100% in mated wild females. However, egg clustering was less frequent (5.6%), despite a higher total number of eggs (n = 10). In contrast, in 2017 and 2018, egg clustering accounted for 38.9% and 23.1% of total egg laying, respectively, including both mated and unmated females (n = 11, 6, respectively). Average egg numbers were lower than those of laboratory females (Table 1).

#### 3.1.3. Life Cycle Under Laboratory Conditions

Morphological and developmental data are presented in Table 2. Larvae are visible within the egg before hatching and emerge bright green with bristles. Within two days, they become melanocytic. Ocelli and darker patterns appear during the second instar. From the third stage onward, colour polymorphism becomes evident. Leaf consumption increases significantly during the fourth and fifth instars. Under optimal laboratory conditions (27 ± 1 °C, 80 ± 5% relative humidity), the full development cycle lasts approximately 30 days. The adult moths have an average lifespan of 36.77 ± 23.62 days, although they can live up to 90 days. Wing damage typically prevents flight after 40 days of life. The sex ratio of the moths was balanced, with 49.8% males and 50.2% females.

#### 3.1.4. Rearing Mortality

Mortality data were analyzed for 2017 and 2019, with data from 2018 excluded due to contamination issues that affected the validity of the results for that year (see Section 3.1.5). In 2017, no mortality was recorded between the second and fifth larval stages, suggesting a stable rearing environment. However, in 2019, mortality occurred across all stages, likely due to a higher rearing density, which could have stressed the larvae.

The first larval stage exhibited the highest mortality rates: 60% in 2017 and 30% in 2019. For the pupal stage, mortality was 10% in 2017 and 7.7% in 2019, while the pre-nymphal stage showed stable mortality around 8% in both years. Some larvae in advanced instars were found dead, partially consumed, or injured, indicative of cannibalism, observed 7 times in 2017 and 58 times in 2019. The increase in observed cases of cannibalism in 2019 may be linked to higher larval densities, which likely contributed to competition for resources and the observed mortality.

#### 3.1.5. Mortality Due to Pathogenic Contamination

**Figure 1:**
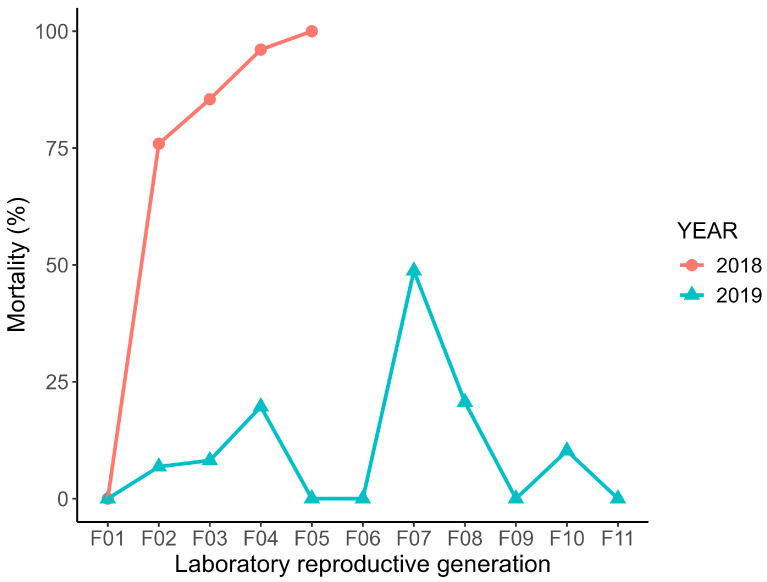
Temporal evolution of pathogenic contamination in laboratory populations (2018–2019). This figure highlights the significant impact of pathogenic contamination on larval survival across different generations of *Eudocima phalonia* in the laboratory during the years 2018 and 2019. The data show the mortality rates associated with the contamination, starting in the F2 generation.

In 2018, contamination began during the F2 generation, with 75.9% mortality. By F4, mortality reached 96%, and no larvae survived beyond L3 in F5 (Figure 1, Table 3). This rapid increase in mortality across successive generations indicates a progressive deterioration of colony health, ultimately leading to complete collapse. Infected larvae exhibited several symptoms, including lethargy, dull coloration, and liquid feces. Dead larvae were often soft or liquefied, found suspended on the walls of the rearing boxes with liquid fecal matter around them. Some larvae that managed to pupate showed signs of internal liquefaction, and approximately 10% of the pupae displayed these symptoms.

A similar contamination occurred in 2019 during F2. Initial mortality was low (<10%) for the first three generations (Table 3). However, mortality increased in later generations, with a first peak observed at F4 (19.7%), followed by a more pronounced peak at F7 (48.7%). After stabilization in F5–F6, a new peak (50%) occurred in F7. Control measures, such as isolating infected larvae and disinfecting the rearing environment, helped limit further outbreaks, but a smaller third peak (10%) was recorded in F10. Unlike 2018, contamination in 2019 did not result in complete colony collapse, suggesting partial control of pathogen spread. Overall, mortality patterns differed markedly between years, with a rapid and irreversible increase in 2018, compared with a more fluctuating and partially controlled dynamic in 2019 (Table 3). These results highlight the strong impact of pathogenic contamination on rearing success and its variability across generations.

### 3.2. Monitoring Network of Eudocima phalonia

#### 3.2.1. Larval Density and Polymorphism in Natural Environments

**Figure 2:**
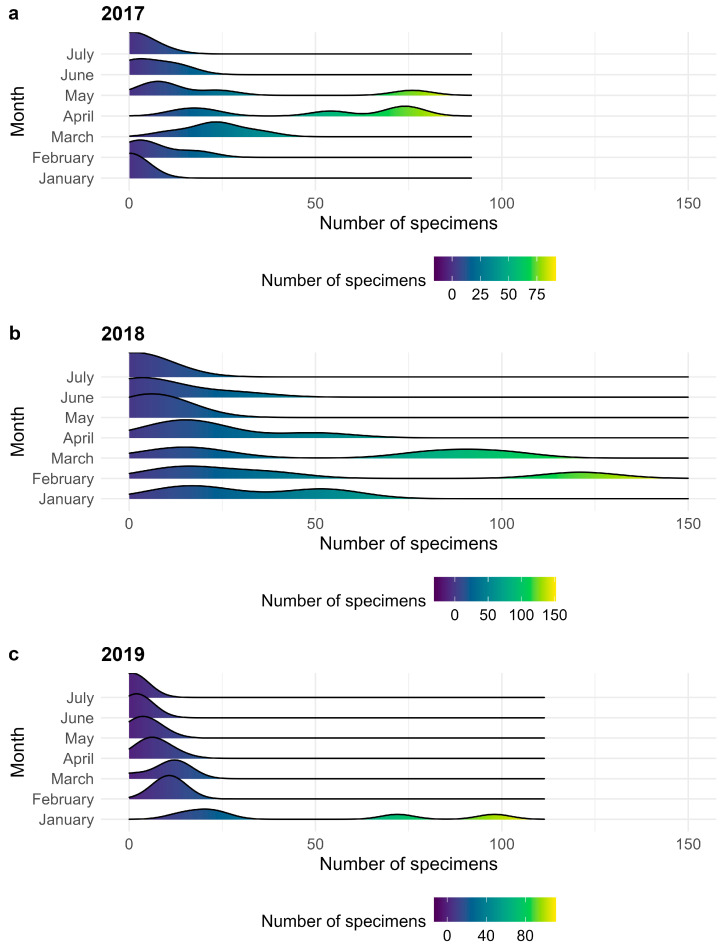
Seasonal variation in *Eudocima phalonia* larval density from 2017 to 2019 (**a**–**c**). Data are presented from January to July, corresponding to the main period of larval activity. Larval abundance declined sharply after July, with fewer than 1–5 individuals recorded per month during the dry season, with adult moths becoming rare after September. Interannual variation is evident: a typical outbreak pattern was observed in 2017, with a peak in April and May, whereas in 2018, larval abundance increased earlier, with a peak occurring between February and March. In 2019, larval abundance remained comparatively low, with no clear outbreak pattern.

In 2017 and 2018, larval outbreaks were recorded and quantified for each year based on n = 543 (2017), n = 944 (2018), and n = 388 (2019) (Figure 2). Larval abundance showed clear seasonal dynamics in all three years, with marked interannual variation in both timing and intensity. In 2017, larval numbers increased from February to March, peaked in April, and declined by late May. This peak corresponded to the highest larval counts recorded during the study period (Figure 2). A similar pattern was observed in 2018; however, the increase in larval abundance occurred earlier, beginning in January and peaking between January and March. In contrast, larval densities were already low in December 2018 despite reported adult-related fruit damage, indicating a temporal mismatch between larval abundance and observed damage. In 2019, a larval peak was observed in January, but overall larval abundance remained lower than in previous years, and no outbreak pattern was detected (Figure 2). After July, larval densities declined sharply in all years, with fewer than five individuals recorded per month during the dry season.

**Figure 3:**
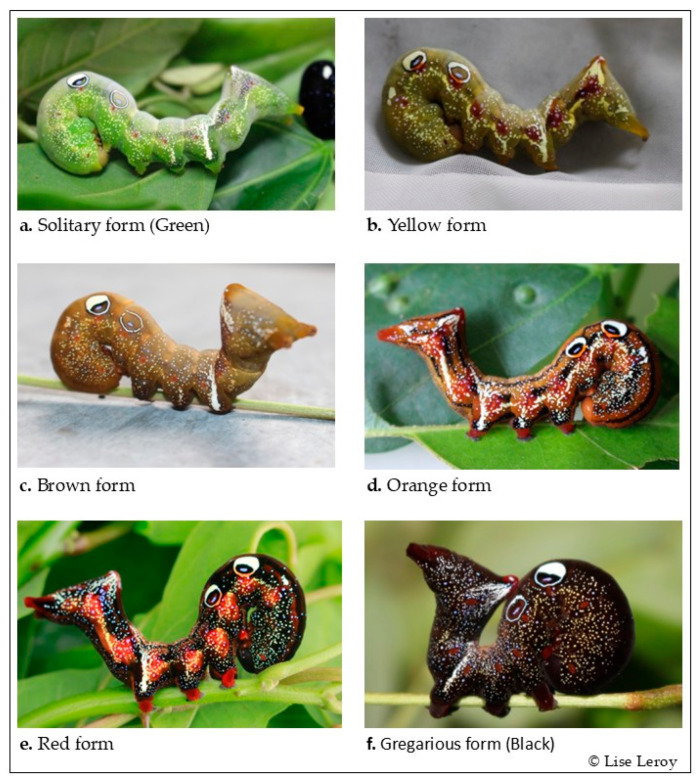
Larval colour form observed in larvae of *Eudocima phalonia*. Representative individuals illustrating the main colour morphs identified in this study (Green, Yellow, Brown, Orange, Red, and Black forms). Photographs include larvae collected from field populations across multiple sites in New Caledonia between 2017 and 2019. These morphotypes were used for subsequent classification and quantitative analysis of polymorphism.

Figure 3 illustrates the different larval colour morphotypes observed in *Eudocima phalonia* (n = 300). The black form (hereafter referred to as the gregarious morph based on field observations) was predominant in all three years, representing the majority of individuals, particularly in 2018 during the outbreak year (Table 4). Specifically, the black form accounted for the highest proportion in 2018, consistent with the peak in larval density observed that year. However, it also remained dominant in 2019 (68.6%), despite the absence of an outbreak, indicating that its occurrence is not exclusively associated with high population density (Table 4). Other larval forms showed interannual variation: the red form was more frequent in 2017 (21.4%), while the brown form was more represented in 2019 (12.1%). Yellow and green forms remained rare across all years (Table 4). Given the unbalanced distribution of morphotypes, these observations should be interpreted descriptively and do not imply causal relationships between coloration and population dynamics.

#### 3.2.2. Relationship Between Pupal Morphology and Sex

Among the 300 pupae analyzed, 165 males (55%) and 135 females (45%) were recorded. Significant differences between sexes were found for pupal length (t = 7.03, *p* = 1.407 × 10^−11^), width (t = 6.72, *p* = 9.196 × 10^−11^), and weight (t = 7.56, *p* = 5.158 × 10^−13^). Overall, female pupae were significantly larger and heavier than male pupae across all measured morphological traits. In particular, females showed greater length (t = 6.76, *p* = 3.842 × 10^−11^) and higher body mass (W = 16595, *p* = 4.635 × 10^−14^). However, no significant difference was observed in developmental duration between sexes (t = 0.92, *p* = 0.3561). This indicates that sexual dimorphism in pupal size and weight does not translate into differences in pupal development time under the experimental conditions used in this study.

#### 3.2.3. Link Between Larval Colour and Pupal Parameters

**Figure 4:**
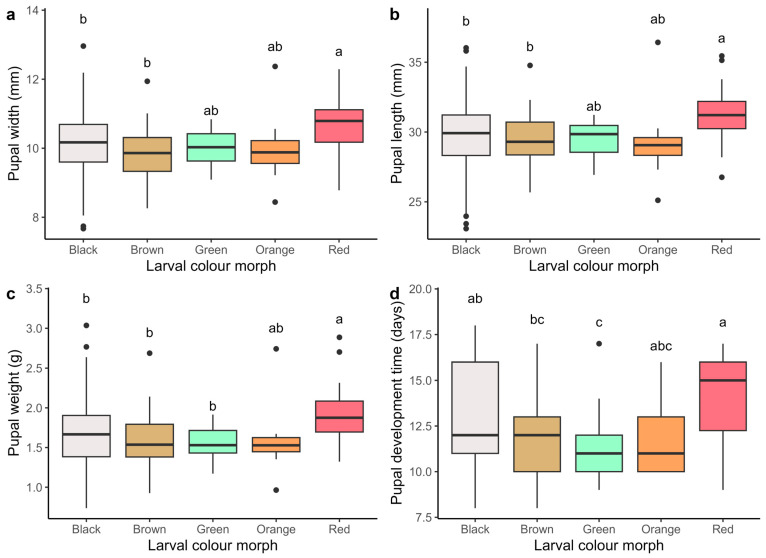
Effect of larval colour morph on pupal morphological and developmental traits. Boxplots show pupal width, length, weight, and developmental time among colour morphs. (**a**) Effect of larval colour on pupal width, (**b**) Effect of larval colour on pupal length, (**c**) Effect of larval colour on pupal weight, (**d**) Effect of larval colour on pupal development time. Different letters above boxes indicate statistically significant differences among groups based on Tukey’s post-hoc test following one-way ANOVA (*p* < 0.05). Groups sharing the same letter are not significantly different, whereas groups with different letters differ significantly.

The sample of 300 field-collected larvae was categorized according to the colour morphs described in Figure 3: green (7%), yellow (0.7%), orange (4.4%), red (7.3%), brown (12.3%), and black (68.3%). Due to the very low representation of the yellow morph, this category was excluded. statistical analyses to avoid biased estimates. ANOVA and post-hoc Tukey tests revealed significant effects of larval colour on pupal weight (*p* = 0.021), length (*p* = 0.0043), width (*p* = 0.0035), and developmental time (*p* = 0.0005). These results indicate that pupal morphological and developmental traits vary significantly among larval colour morphotypes. A combined linear model confirmed the effect of larval coloration on the set of measured traits (*p* = 0.00025). However, effect sizes varied among morphs, with red larvae showing the most distinct deviation from other groups, while black morphs exhibited higher intra-group variability (Figure 4). A Chi-square test showed no association between larval colour and sex ratio (χ^2^ = 6.09, df = 5, *p* = 0.298), indicating that larval coloration is not associated with sex determination under the conditions of this study. These associations are based on observational field data and should not be interpreted as evidence of causal relationships between larval coloration and developmental performance.

## 4. Discussion

### 4.1. Rearing Conditions of Eudocima phalonia

Although the laboratory rearing was not continuous over the three-year period, successful reproduction was achieved over up to 11 successive generations in 2019, yielding more than 3000 individuals. This demonstrates that *E. phalonia* can be maintained under controlled laboratory conditions, although with variable reproductive success across years.

The pre-oviposition period recorded in this study (8–13 days) is consistent with previous observations in *E. materna* [17] and *E. phalonia* [18], and supports the general assumption that mating occurs within the first days post-emergence [6]. However, this remains an indirect inference, as mating events were not directly observed in the present study. Some authors report that eggs are already fully developed at adult emergence [18], whereas others suggest continued maturation after emergence [6], indicating that this aspect of reproductive biology may still vary depending on environmental conditions.

Sex ratio and developmental parameters observed in our study are broadly consistent with those reported by Cochereau [6]. However, direct comparisons with field populations should be made cautiously, as controlled temperature, photoperiod, and humidity conditions likely optimized developmental rates and reduced environmental variability compared with natural conditions.

Statistical analyses indicate that pupal morphological traits (length, width, and weight) are significantly greater in females than in males. These differences may allow partial sex differentiation based on pupal morphology. However, no causal relationship can be inferred between size and developmental duration, as no significant difference in development time was observed between sexes. This suggests that developmental duration is likely influenced more strongly by abiotic conditions (temperature, photoperiod) and internal physiological regulation than by final body size [19,20,21,22], as previously suggested in Lepidoptera [23].

The rearing conditions, particularly density and continuous laboratory maintenance, may have influenced larval behavior and phenotype expression. A high proportion of larvae exhibited dark or red coloration in both laboratory and field conditions. In Lepidoptera, such phenotypic variation has sometimes been associated with density-dependent responses or environmental plasticity [24,25]. Factors such as female fecundity, resource abundance, and predation pressure may also influence these patterns [26,27]. However, in the present study, these associations remain correlative and cannot be interpreted as direct adaptive mechanisms.

Egg clustering behavior was observed in both laboratory and field populations, although its frequency varied across years. Notably, in 2019, clustering was lower in wild populations (5.6%) compared with laboratory-reared individuals (28.8%) for mated and unmated females. This difference may reflect environmental or population context effects, but also potentially sampling constraints or differences in female physiological status at capture. Females were observed to lay both solitary and clustered eggs within the same oviposition period, indicating substantial intra-individual variability.

Overall, the observed variation in reproductive traits and egg-laying strategies suggests a high degree of plasticity in *Eudocima phalonia*. However, the present dataset does not allow formal testing of fitness consequences or adaptive value of these traits, and interpretations regarding outbreak dynamics should therefore remain cautious and considered as hypotheses for future experimental validation.

### 4.2. Mortality in Rearing

High mortality in early larval stages was likely associated with a combination of nutritional limitations and crowding in rearing containers, as previously reported in other Lepidoptera species [28]. However, these factors were not experimentally isolated in the present study, and their relative contribution cannot be quantified.

Pre-nymphal mortality was mainly observed when individuals failed to successfully complete metamorphosis or were inadvertently disturbed during routine handling. In some cases, insufficient availability of leaf material for cocoon construction may also have contributed to mortality. Additionally, a proportion of smaller larvae exhibited premature pupation, resulting in non-viable pupae, likely associated with suboptimal nutritional conditions.

Pupal mortality, particularly during colder periods, may have been influenced by environmental conditions such as temperature fluctuations and possible desiccation. However, given that emergence failures were not systematically linked to measured humidity thresholds, the role of desiccation remains speculative. Previous work indicates that low humidity alone does not necessarily prevent successful emergence in this species [6]. The possibility of diapause was considered but not evidenced in this study. In tropical Lepidoptera, diapause or quiescence can occur at different life stages and is generally less well characterized than in temperate species [29]. Cochereau [6] suggested that part of the *E. phalonia* population may persist through periods of reduced reproductive activity and extended development during winter. Such a quiescent state may allow insects to temporarily suspend development and resume activity under favorable conditions [23]. For example, at temperatures below 16 °C, *E. salaminia* exhibits reduced mating and oviposition activity [30]. However, no direct evidence of diapause or physiological arrest was observed in the present study.

Cannibalism, although not a dominant mortality factor, was mainly recorded in the 4th and 5th larval instars. This behavior has also been reported in other noctuid species such as *Spodoptera frugiperda* [31,32,33], where it is generally considered a density-dependent response potentially linked to competition for resources. Cannibalism may also provide short-term nutritional advantages under stressful conditions [34]. In the present study, it accounted for approximately 4.5% of total mortality, which remains low compared with mortality associated with pathogenic contamination (96.07%).

Pathogenic contamination likely originated from external sources, including fresh host plant material or newly introduced field-collected larvae. The gregarious nature of larval development may have facilitated transmission under laboratory conditions [35,36], and cannibalism may have further contributed to horizontal pathogen spread according to E. Herniou (personal communication, 2018). Isolation of infected individuals allowed the identification of eight bacterial and three fungal strains; however, their pathogenicity and relative virulence were not experimentally tested in this study and require further investigation based on unpublished data from L. Leroy (2021) [8]. Biological control agents such as *Bacillus thuringiensis* are widely used against agricultural pests worldwide [37], including species closely related to *Eudocima materna* [38,39]. Other bacterial agents, such as *Saccharopolyspora spinosa* or *Photorhabdus luminescens*, have also been reported as effective against larvae of *E. materna* [38,39]. Fungal pathogens have been documented as well, although they are generally considered less effective against *E. phalonia* compared with bacterial toxins [38,39]. Viral agents, including baculoviruses, also represent promising candidates for biological control [40,41]. Vertical transmission through asymptomatic carriers could potentially contribute to long-term population regulation, as suggested by phylogenetic studies showing long-term host specialization in these viruses [42]. Nevertheless, these biological control perspectives remain speculative in the absence of direct experimental validation on the studied populations.

### 4.3. Larval Polymorphism in Wild Populations

Our results indicate that larval coloration is significantly associated with several pupal morphological traits, while no relationship was detected with adult sex. Larval polymorphism is widely documented in Lepidoptera [19,35,43,44], including in *E. materna* and *E. phalonia* [6,18,45]. In these species, black and brown forms are commonly reported [9,45], although green, yellow, and intermediate forms have also been described in some populations [46,47]. In many Lepidoptera, larval coloration has been associated with density-dependent and ecological strategies. Solitary forms are generally more frequent at low population densities and are often described as investing more in camouflage and extended development [6,43]. In contrast, gregarious forms are frequently associated with darker pigmentation, increased feeding activity, and reduced developmental time [19]. However, these general patterns are species-dependent and may not be directly transferable to *E. phalonia*.

In the present study, darker larvae were predominant, particularly black morphs, while red forms showed stronger associations with certain pupal traits, including larger size and longer developmental duration. This pattern differs from observations reported in other Noctuoidea (e.g., *Spodoptera*, *Mamestra*), where darker forms are more commonly associated with reduced developmental time and smaller body size. These differences suggest that colour–trait associations in *E. phalonia* may be more complex than a simple gregarious–solitary dichotomy and may involve additional environmental or physiological factors.

However, these interpretations must be made with caution. The dataset is strongly unbalanced, with black morphs representing 68.3% of individuals, while other morphs were comparatively rare. In addition, larvae were collected in the field at different developmental stages and under heterogeneous environmental conditions. These factors limit direct comparisons of developmental duration and may introduce sampling bias.

Environmental variables such as density, temperature, and host plant quality have been shown to influence larval coloration in Lepidoptera [35,43,48,49]. In particular, melanization has been associated with thermoregulation and increased UV absorption in some species [43,50,51]. Nevertheless, the present study does not allow discrimination between genetic, environmental, or plastic contributions to larval coloration, and the observed associations should therefore be considered exploratory.

### 4.4. Outbreak and Non-Outbreak Dynamics of Eudocima phalonia

Larval monitoring between 2017 and 2019 revealed two years characterized by high larval abundance (2017 and 2018) and one year with comparatively lower and non-sustained population peaks (2019). The 2017 dynamics were broadly consistent with previous observations in New Caledonia [6], whereas the 2018 increase occurred earlier in the season. These differences suggest that outbreak timing may vary interannually, although the underlying drivers remain difficult to disentangle based on the available data.

Outbreak dynamics in Lepidoptera are generally considered to result from multiple interacting factors, including climatic variability, host plant availability, and dispersal processes [52,53,54]. In the present study, transitions from more aggregated larval distributions to higher local densities were observed during peak periods. However, a direct causal link between egg clustering, larval gregariousness, and outbreak initiation cannot be established from the current dataset.

Cochereau [6] proposed that rainfall anomalies, particularly deficits exceeding 50% prior to outbreak years, may be associated with increased population levels in subsequent seasons. In this context, 2016 was characterized by unusually high temperatures followed by drought conditions in 2017, while rainfall patterns in early 2017 coincided with increased larval abundance later in the year. A similar sequence was observed between late 2017 and early 2018. Although these patterns are consistent with the hypothesis that climatic variability may influence population dynamics, no statistical model was developed in this study to formally test these relationships. More generally, large-scale climatic phenomena such as the El Niño–Southern Oscillation (ENSO) have been suggested to influence insect population fluctuations in tropical regions [55], but their specific role in *E. phalonia* remains unquantified.

Fruit phenology may also contribute to temporal variation in population abundance. In New Caledonia, fruit availability begins with lychee in December and extends until late winter (e.g., citrus species). This extended resource availability may support successive generations and influence adult reproduction and larval survival. However, the present study did not include quantitative measurements of fruit availability or host plant abundance, limiting direct assessment of this factor.

Egg clustering rates varied between 5.6% and 38.9% across years. Such variation may contribute to spatial heterogeneity in larval density, potentially affecting visibility to natural enemies such as predators and parasitoids [56]. However, the role of natural enemies was not measured in this study, and their contribution to population regulation remains speculative.

Interestingly, differences in the proportion of colour larvae forms were observed between years. In 2018, black larvae were predominant in 2017, while in 2019, black morphs remained frequent despite reduced outbreak intensity. These observations suggest that larval coloration alone cannot be directly used as a proxy for outbreak status, and that multiple ecological and environmental factors likely interact to determine phenotypic distribution. This can suggest two possibilities: (1) the gregarious form confers biological advantages regardless of outbreaks, or (2) return to solitary behavior is delayed, as observed in *Schistocerca gregaria* [57].

Overall, the results suggest that *Eudocima phalonia* populations exhibit strong inter-annual variability in abundance and structure. However, the available data do not allow the identification of a single primary driver of outbreak dynamics. Population fluctuations are likely the result of interacting environmental and biological factors, and further long-term and experimental studies would be required to disentangle these mechanisms.

## 5. Conclusions

The successful laboratory rearing of *Eudocima phalonia* was achieved through controlled environmental conditions, allowing the completion of multiple generations and the production of sufficient individuals for biological and ecological studies. However, rearing success varied across years, indicating that some aspects of the species’ biology remain sensitive to environmental or methodological factors that were not fully controlled in this study. A better understanding of the species’ biology is still required to identify and quantify the main factors influencing its development, reproduction, and population dynamics. In particular, larval coloration appears to be associated with several developmental and morphological traits. However, these associations are based on observational data and do not allow causal inference regarding underlying genetic or environmental mechanisms.

Although phenotypic variation was observed across laboratory and field conditions, no direct measurements of fitness components (e.g., survival to reproduction, fecundity under controlled conditions across larval forms) were performed in this study. Consequently, any interpretation in terms of adaptive value should remain speculative. Larval polymorphism may reflect a combination of environmental plasticity and physiological regulation, as commonly observed in Lepidoptera, rather than a single adaptive strategy.

Accelerated development and variation in body size likely represent multiple responses to environmental conditions rather than a single optimized strategy. The present study does not allow ranking of these strategies in terms of fitness or adaptive advantage, but it highlights the variability of developmental responses in *E. phalonia* under both laboratory and field conditions.

From an applied perspective, the establishment of a reproducible rearing protocol represents an important step toward experimental work on this species. Such a system may facilitate future studies on biology, ecology, and potential control strategies. Although integrated pest management approaches combining biological, environmental, and behavioral components remain to be fully developed for *E. phalonia*, the present work provides baseline biological and methodological data that can support future applied research. Further studies are needed, particularly those addressing the mechanistic basis of larval polymorphism and its relationship to environmental variation and population dynamics, in order to better predict and manage outbreak events.

## Figures and Tables

**Table 1:** Egg-laying performance of reared and wild *Eudocima phalonia* females (2017–2019). Pre-oviposition time (days), laying time (days), and average number of eggs laid are presented. Values are expressed as mean ± standard deviation (SD). Calculations are based only on females that laid eggs (fertilized or not) and do not include females that died without laying eggs. The sample sizes for reared females are 9 in 2017 (4 mated, 5 unmated), 9 in 2018 (4 mated, 5 unmated), and 30 in 2019 (11 mated, 19 unmated). The sample sizes for wild females are 11 in 2017 (5 mated, 6 unmated), 6 in 2018 (4 mated, 2 unmated), and 10 in 2019 (10 mated, 0 unmated). Pre-oviposition and laying times were recorded as the time from emergence to the first egg deposition for mated and unmated females.

	2017		2018		2019	
	Reared	Wild	Reared	Wild	Reared	Wild
**Pre-oviposition time**						
Mated Female	11.70 ± 1.50	6.20 ± 1.90	10.80 ± 2.00	3.50 ± 3.60	11.00 ± 1.20	2.70 ± 2.26
Unmated Female	11.00 ± 2.10	7.00 ± 2.00	10.25 ± 1.80	5.00 ± 1.40	11.00 ± 1.20	0
**Laying time**						
Mated Female	3.50 ± 3.30	4.00 ± 3.60	7.80 ± 5.40	6.00 ± 2.90	11.90 ± 4.90	7.60 ± 5.40
Unmated Female	5.60 ± 2.30	8.00 ± 3.60	6.70 ± 2.05	7.50 ± 4.90	10.40 ± 4.80	0
**Average of eggs laid**						
Mated Female	106.20 ± 102.30	53.80 ± 31.36	303.20 ± 226.90	278.50 ± 122.90	363.90 ± 234.00	245.10 ± 216.40
Unmated Female	238.00 ± 100.90	288.60 ± 154.20	225.20 ± 157.70	464.50 ± 267.90	442.30 ± 224.20	0

**Table 2:** Morphological traits and developmental duration of *Eudocima phalonia* under laboratory conditions. Development time is given in days. Values are expressed as mean ± standard deviation (SD). Sample size for each developmental stage is n = 15. Morphological data for each stage of development: length and width are given in millimeters, and weight (referring to live mass) is given in grams.

Developmental Stage	Development Time (Days)	Morphological Characteristics		
		Length (mm)	Width (mm)	Weight (g)
Egg	3.60 ± 0.89	-	-	-
1st larval stage	3.46 ± 0.66	4.46 ± 0.60	-	<0.001
2nd larval stage	3.30 ± 0.63	15.06 ± 0.97	-	0.020 ± 0.001
3rd larval stage	3.00 ± 0.58	35.50 ± 2.67	-	0.324 ± 0.034
4th larval stage	2.30 ± 0.63	61.37 ± 6.94	-	1.538 ± 0.367
5th larval stage	2.77 ± 1.16	76.25 ± 7.85	-	3.393 ± 0.437
Pupae	12.73 ± 2.69	29.76 ± 2.15	10.12 ± 0.87	1.656 ± 0.384

**Table 3:** Mortality of *Eudocima phalonia* larvae due to pathogenic contamination in 2018 and 2019. This table shows the total number of larvae and the corresponding number of dead larvae for each generation in the years 2018 and 2019. The mortality rate (%) can be derived from the ratio of dead larvae to the total number of larvae in each generation.

Generation	2018			2019		
	Number of Larvae	Number of Dead Larvae	Mortality Rate (%)	Number of Larvae	Number of Dead Larvae	Mortality Rate (%)
F1	17	0	0	584	0	0
F2	270	205	75.9	190	13	6.8
F3	268	229	85.5	330	27	8.2
F4	178	171	96	376	74	19.7
F5	80	80	100	540	0	0
F6	-	-	-	62	0	0
F7	-	-	-	363	177	48.7
F8	-	-	-	368	76	20.7
F9	-	-	-	30	0	0
F10	-	-	-	312	32	10.3
F11	-	-	-	11	0	0

**Table 4:** Proportion of larval colour form of larvae of *Eudocima phalonia* recorded in field populations from 2017 to 2019. Percentages are based on n = 300 larvae collected across all sampling sites and dates. Larvae were classified into six morphotypes (Green, Yellow, Orange, Brown, Red, and Black) according to visual criteria described in Figure 3. Due to uneven sample sizes among morphotypes, particularly the dominance of the black morph, these data are presented for descriptive purposes only.

Colour Form of Larvae	2017	2018	2019
Green	6.8	5.4	8.7
Yellow	1.2	0.4	3.8
Brown	6.6	10.8	12.1
Orange	4.8	2.7	5.6
Red	21.4	2.4	1.2
Black	59.2	78.3	68.6

## Data Availability

The dataset generated during and/or analyzed during the current study are available from the corresponding author on reasonable request.

## References

[1] Porter J.H., Parry M.L., Carter T.R. (1991). The potential effects of climatic change on agricultural insect pests. Agric. For. Meteorol..

[2] Fuhrer J. (2003). Agroecosystem responses to combinations of elevated CO_2_, ozone, and global climate change. Agric. Ecosyst. Environ..

[3] Estay S.A., Lima M., Labra F.A. (2009). Predicting insect pest status under climate change scenarios: Combining experimental data and population dynamics modelling. J. Appl. Entomol..

[4] Gagnon A.-È., Bourgeois G., Bourdages L., Grenier P., Blondlot A. (2019). Impact of climate change on *Ostrinia nubilalis* (Lepidoptera: Crambidae) phenology and its implications on pest management. Agric. For. Entomol..

[5] Risbec J. (1942). Observations on the Insects of the Plantations in New Caledonia—Observations Sur Les Insectes des Plantations en Nouvelle-Calédonie.

[6] Cochereau P. (1977). Biology and Ecology of Fruit-Piercing Moth Populations *Othreis fullonia* Clerck (Lepidoptera: Noctuidae, Catocalinae) in New Caledonia. Ph.D. Thesis.

[7] Waterhouse D.F., Norris K.R. (1987). Biological Control: Pacific Prospects.

[8] Leroy L., Mille C.G., Fogliani B. (2021). The common fruit-piercing moth in the Pacific region: A survey of the current state of a significant worldwide economic pest, *Eudocima phalonia* (Lepidoptera: Erebidae), with a focus on New Caledonia. Insects.

[9] Hargreaves E. (1936). Fruit-piercing Lepidoptera in Sierra Leone. Bull. Entomol. Res..

[10] Golding F.D. (1945). Fruit-piercing Lepidoptera in Nigeria. Bull. Entomol. Res..

[11] Balikai R.A., Kotikal Y.K., Prasanna P.M. (2009). Status of pomegranate pests and their management strategies in India. Acta Hortic..

[12] Jayanthi P.D.K., Verghese A., Nagaraju D.K., Jhansi R. (2010). Studies on the possibility of managing fruit sucking moth, *Eudocima materna* (Lepidoptera: Erebidae) using feeding repellents. Pest Manag. Hortic. Ecosyst..

[13] Fay H.A.C., Halfpapp K.H. (2006). Synthetic Fruit Piercing Moth Attractant. Australian Patent.

[14] Mallikarjun K.R.M., Thippaiah M., Raghavendra A., Sharma J., Chakravarthy A.K. (2019). Role of fruit volatiles and sex pheromone components in mate recognition in fruit-piercing moth *Eudocima materna*. J. Entomol. Zool. Stud..

[15] Kumar K., Lal S.N. (1983). Studies on the biology, seasonal abundance and host–parasite relationship of fruit-sucking moth *Othreis fullonia*. Fiji Agric. J..

[16] Muniappan R., Bamba J., Cruz J., Reddy G.V.P. (2004). Biology, rearing and field release on Guam of *Euplectrus maternus*, a parasitoid of the fruit-piercing moth *Eudocima fullonia*. BioControl.

[17] Younghusband J.E. (1979). The life history and description of the fruit-piercing moth *Eudocima materna*. Rhod. Agric. J..

[18] Baptist B.A. (1944). The fruit-piercing moth (*Othreis fullonia*) with special reference to its economic importance. Indian J. Entomol..

[19] Aguillon D.J., Medina C., Velasco L.R.I. (2015). Effects of larval rearing temperature and host plant condition on development and coloration of *Spodoptera exempta*. J. Environ. Sci. Manag..

[20] Akiyama K., Nishida T. (2013). Highly enhanced larval growth during the cold season mediated by basking behavior. Entomol. Sci..

[21] Chen C., Xia Q.-W., Fu S., Wu X.-F., Xue F.-S. (2013). Effect of photoperiod and temperature on pupal diapause. Bull. Entomol. Res..

[22] Qureshi M.H., Murai T., Yoshida H., Shiraga T., Tsumuki H. (1999). Effects of photoperiod and temperature on development and diapause. Jpn. J. Appl. Entomol. Zool..

[23] Saunders D.S. (2019). Dormancy, diapause, and insect photoperiodism. Annu. Rev. Entomol..

[24] Stamp N.E. (1980). Egg deposition patterns in butterflies. Am. Nat..

[25] Hunter A.F. (2000). Gregariousness and repellent defenses in phytophagous insects. Oikos.

[26] Clark B.R., Faeth S.H. (1998). Evolution of egg clustering in butterflies. Evol. Ecol..

[27] Matsumoto K., Ito F., Tsubaki Y. (1993). Egg cluster size variation. Res. Popul. Ecol..

[28] Fantinou A.A., Tsitsipis J.A. (1999). Effect of larval density on development. J. Appl. Entomol..

[29] Denlinger D.L. (1986). Dormancy in tropical insects. Annu. Rev. Entomol..

[30] Sands D.P.A., Schotz M., Bourne A.S. (1991). Effects of temperature on development. Bull. Entomol. Res..

[31] Chapman J.W., Williams T., Escribano A., Caballero P., Cave R.D., Goulson D. (1999). Cannibalism and virus transmission. Ecol. Entomol..

[32] Ferguson H.J., Eaton J.L., Rogers C.E. (1997). Larval density effects on lipid reserves. J. Agric. Entomol..

[33] Da Silva C.S.B., Parra J.R.P. (2013). New method for rearing *Spodoptera frugiperda*. Rev. Bras. Entomol..

[34] Joyner K., Gould F. (1985). Developmental consequences of cannibalism. Ann. Entomol. Soc. Am..

[35] Goulson D., Cory J.S. (1995). Responses to crowding and disease resistance. Oecologia.

[36] Hochberg M.E. (1991). Viruses as costs to gregarious behavior. Oikos.

[37] Beegle C.C., Yamamoto T. (1992). History of *Bacillus thuringiensis*. Can. Entomol..

[38] Magar P.N. (2014). Seasonal Incidence and Management of Fruit Sucking Moth. Master’s Thesis.

[39] Kulkarni S.K., Patil S.K., Guru P.N. (2017). Host specificity and management of fruit sucking moth. Pest Manag. Hortic. Ecosyst..

[40] Valicente F.H. (2019). Entomopathogenic viruses. Natural Enemies of Insect Pests.

[41] Moscardi F., de Souza M.L., de Castro M.E.B., Szewczyk B. (2011). Baculovirus pesticides. Microbes and Microbial Technology.

[42] Herniou E.A., Olszewski J.A., O’Reilly D.R., Cory J.S. (2004). Coevolution of baculoviruses and hosts. J. Virol..

[43] Gunn A. (1998). Determination of larval coloration. Entomol. Exp. Appl..

[44] Tojo S. (1991). Variation in phase polymorphism. Appl. Entomol. Zool..

[45] Tryon H. (1924). Orange piercing moths. Qld. Agric. J..

[46] Comstock J.A. (1963). Fruit piercing moth of Samoa. Can. Entomol..

[47] Maddison P.A. (1982). Fruit Piercing Moths.

[48] Anazonwu D.L., Johnson S.J. (1986). Effects of host and density. Environ. Entomol..

[49] Yamasaki A., Shimizu K., Fujisaki K. (2009). Effect of host plant part. Ann. Entomol. Soc. Am..

[50] Altstein M., Ben-Aziz O., Gazit Y. (1994). PBAN and colour polymorphism. J. Insect Physiol..

[51] Goulson D. (1994). Larval melanization and thermoregulation. Heredity.

[52] Harvey A.W., Mallya G.A. (1995). Predicting outbreak severity. Bull. Entomol. Res..

[53] Holt J., Mushobozi W.L., Tucker M.R., Venn J.F. (1999). Modelling population dynamics. Workshop Proceedings.

[54] Westbrook J.K., Nagoshi R.N., Meagher R.L., Fleischer S.J., Jairam S. (2015). Modeling migration. Int. J. Biometeorol..

[55] Delcroix T., Lenormand O. (1997). ENSO signals near New Caledonia. Oceanol. Acta.

[56] Sillén-Tullberg B., Leimar O. (1988). Evolution of gregariousness. Am. Nat..

[57] Roessingh P., Simpson S.J. (1994). Behavioural phase change. Physiol. Entomol..

